# The Exploitation of a Hempseed Byproduct to Produce Flavorings and Healthy Food Ingredients by a Fermentation Process

**DOI:** 10.3390/microorganisms9122418

**Published:** 2021-11-23

**Authors:** Lorenzo Nissen, Flavia Casciano, Elena Babini, Andrea Gianotti

**Affiliations:** 1 CIRI (Interdepartmental Centre of Agri-Food Industrial Research), *Alma Mater Studiorum*—University of Bologna, P.za Goidanich 60, 47521 Cesena, Italy; lorenzo.nissen@unibo.it (L.N.); elena.babini2@unibo.it (E.B.); 2 DISTAL (Department of Agricultural and Food Sciences), *Alma Mater Studiorum*—University of Bologna, V. le Fanin 44, 40127 Bologna, Italy; flavia.casciano2@unibo.it; 3 DISTAL (Department of Agricultural and Food Sciences), Campus of Food Sciences, *Alma Mater Studiorum*—University of Bologna, P.za Goidanich 60, 47521 Cesena, Italy

**Keywords:** *Futura 75*, scent, multivariate analysis, hexanoic acid, off-odors, bioactives

## Abstract

Following the One Health principles in food science, the challenge to valorize byproducts from the industrial sector is open. Hemp (*Cannabis sativa* subsp. *sativa*) is considered an important icon of sustainability and as an alternative food source. Hemp seed bran, in particular, is a byproduct of industrial hemp seed processing, which is not yet valorized. The success, and a wider market diffusion of hemp seed for food applications, is hindered by its unpleasant taste, which is produced by certain compounds that generally overwhelm the pleasant bouquet of the fresh product. This research concerns the exploration of hemp seed bran through fermentation using beneficial lactobacilli, focusing on the sensorial and bioactive traits of the products when they are subjected to bacterial transformation. By studying of the aromatic profile formation during the fermentation process the aim was to modulate it in order to reduce off-odors without affecting the presence of healthy volatile organic compounds (VOCs). Applying multivariate analyses, it was possible to target the contribution of processing parameters to the generation of flavoring and bioactive compounds. To conclude, the fermentation process proposed was able to reduce unpleasant VOCs, whilst at the same time keeping the healthy ones, and it also improved nutritional quality, depending on time and bacterial starters. The fermentation proposed was a sustainable biotechnological approach that fitted perfectly with the valorization of hemp byproducts from the perspective of a green-oriented industrial process that avoids synthetic masking agents.

## 1. Introduction

Foods formulated with hemp (*Cannabis sativa* subsp. *sativa*) are being increasingly accepted by consumers, and the global sector is expected to generate almost USD 5 billion by 2026 with a CAGR (compound annual growth rate) of around 6.2% between 2019 and 2026 [1]. The edible part of hemp most used in the food industry is the seed, which is a food; it is healthy (not allergenic, free of cholesterol, and rich in polyphenols), nutritious (with high and balanced protein content, fibers, minerals, and vitamins), and functional (rich in bioactive compounds such as flavonoids, terpenes, and organic acids) [2,3,4]. The part of the hemp seed that has the highest commercial value is the oil. During food industrial processes two other worthy products are obtained, hemp seed flour and the proteinaceous cake. The flour, resulting from the grinding of hemp seeds, is intended for human consumption and is rich in bioactive compounds [5], while the cake is traditionally dedicated to animal feed, even if applications in foods have been entering the market recently. From this process, the derived bran is a byproduct, potentially of high, value that is usually discarded. Considering the rising demand for hemp foods and also the pursuance of higher levels of sustainability, the bran merits further food applications. Besides, the important role of hemp flour is increasing, a specific reason for this could be that of the potential prebiotic properties of hemp seed bran (HSB), as we recently have demonstrated [6]. However, the major fact that hinders hemp seed bran distribution is the unpleasant taste ruled by amines and organic acids that, especially during physical transformation, comes to cover the opposite, pleasant bouquet of unprocessed material. The industry up until today has found solutions through invasive practice that mask the stinky traits but reduce the nutritional quality [7]. To maintain a proper balance of nutritional and sensorial features we are proposing fermentation with beneficial lactobacilli, which is able not solely to confer a new aromatic profile, but even to augment the content of nutritional compounds and bioactives as well as their bioavailability [8]. *Lacticaseibacillus rhamnosus*, *Lactiplantibacillus plantarum*, and *Limosilactobacillus fermentum* harbor different alpha-glucosidases that make them ideal to ferment plant-based stuffs [9,10]; besides, strains of these species are considered probiotics, such as *Lacticaseibacillus rhamnosus* GG (LGG) [11], *Lactiplantibacillus plantarum* K10 [12], and *Limosilactobacillus fermentum* ME-3 [13]. Thus, in this work, with the aim of valorizing a hemp industrial byproduct, we fermenteded hemp seed bran with different lactobacilli and their pool, and we characterized their effect on volatilome as contributions given to flavoring and bioactive compounds.

## 2. Materials and Methods

### 2.1. Hemp Seed Bran Preparation

HSB, a byproduct remaining after the mechanical pressing of hemp seeds and subsequent grinding and sieving, was supplied by a local company (Hemp Positive World, Cesena, Italy). The original hemp variety was Futura 75. A volume of 30 mL HSB/water suspensions (1:6 *w/v*) were sterilized in independent 50 mL plastic tubes (121 °C and 1 bar for 15 min), then used as substrates for bacterial fermentations. 

### 2.2. Microbial Strains and Culture Conditions

All microbial strains tested belonged to the microbial collection of DISTAL (Dept. of Agricultural and Food Sciences), University of Bologna (Bologna, Italy) and were previously isolated from plant-based products and extensively studied [10,14,15]. *Lactiplantibacillus plantarum* 98b, *Limosilactobacillus fermentum* PRLF, and *Lacticaseibacillus rhamnosus* C1112 (used for hemp bran fermentation) were cultured from 30% (*v/v*) glycerol stocks stored at −80 °C and were propagated, cultured, and enumerated on de Man Rogosa Sharpe (MRS) broth (Oxoid, Thermo Fisher Scientific, Waltham, MA, USA) at 37 °C in microaerophilic conditions. An oxygen catalyst (Oxoid, Thermo Fisher Scientific, USA) was applied to the jars for at least 48 h.

### 2.3. Fermentations

The hemp bran samples were fermented independently by *Lacticaseibacillus rhamnosus* C1112 (Lr), *Lactiplantibacillus plantarum* 98b (Lp), *Limosilactobacillus fermentum* PRLF (Lf), and by a bacterial mix (m) containing equal proportions of the aforementioned strains. Cell load of the inoculated bacteria was standardized at 7 Log_10_ cells/mL by spectrophotometric means based on plate counts (Figure S1). Bacterial cells were centrifugated and resuspended two times in sterile water before being added to sterile HSB/water suspensions (1:6 *w/v*), whose fermentations were conducted in 30 mL tubes that were incubated aerobically at 37 °C for up to 72 h to obtain fermented hempseed bran samples. Each duplicate of a time point sample was made in distinct tubes. Sterile HSB/water suspensions (1:6 *w/v*) with no addition of bacteria were used as controls. For each inoculated sample (Lr, Lp, Lf, and m), sampling was performed after 6, 24, 48, and 72 h. Analyses were performed with respect to bacterial quantifications, pH, and VOCs (volatile organic compounds) characterization. In this work we populated our dataset using results obtained from biological duplicates of the experiment, which were performed at different times, and technical duplicates performed at the same time (*n* = 4). Analyses were made with analytical duplicates (VOCs and plate count) and analytical triplicates (pH and qPCR).

### 2.4. Bacterial Cell Measurement

For all bacteria, 1 mL of each sample was aseptically transferred into a sterile glass tube with 9 mL of physiological solution (9 g/L NaCl) and then serially diluted (1:10) and plated in duplicates. Lactobacilli and their pool were counted on MRS agar medium (Oxoid, Thermo Fisher Scientific, USA) after having been incubated for at least 24 h at 37 °C. Bacterial quantification was obtained by both culture-dependent and culture-independent protocols. The first consisted of plating on selective MRS agar the serial dilutions of the samples previously obtained (NaCl 0.9%, *w/v*), then incubating them for 24 h aerobically at 37 °C. Values were expressed as Log_10_ CFU/mL. Quantifications by plate count were obtained from the samples in analytical duplicates. Culture-independent quantifications were obtained by qPCR with the SYBR Green I chemistry, Eubacteria degenerated primers were applied as previously described (Eub518-R: 5′- ATTACCGCGGCTGCTGG-3′ and Eub338-F: 5′-ACTCCTACGGGAGGCAG-3′) [16]. Genetic standards were prepared from relative PCR amplicons obtained from the DNA of samples of HSB/water suspensions (1:6 *w/v*) that had been fermented for 72 h by the pool of lactobacilli, using a Pro-Flex PCR apparatus (Applied Biosystem, Foster City, CA, USA). The standards were extracted and purified using the DNA purification kit as described previously [6,9,10,15]. Bacterial DNA extraction and qPCR reactions were performed according to previous protocols that employed a RotorGene 6000 (Qiagen, Hilden, Germany) [6,9,10,15]. Quantification results were reported as the means of the values of the two techniques employed and expressed as Log_10_ cells/mL [6,9,10,15]. Quantifications by qPCR were obtained from the samples in analytical triplicates.

### 2.5. pH Measurement

The pH was determined with a pH meter (Crison, Alella, Spain) at 20 °C, appropriately calibrated with three standard buffer solutions at pH 9.21, pH 4.00, and pH 2.00. The pH values were measured from the samples in analytical triplicates at three different times to monitor the fermentation.

### 2.6. Solid-Phase Microextraction–Gas Chromatography–Mass Spectrometry (SPME–GC–MS)

Evaluation of VOCs was carried out on an Agilent 7890A Gas Chromatograph (Agilent Technologies, Santa Clara, CA, USA) coupled with an Agilent Technologies 5975 mass spectrometer operating in the electron impact mode (ionization voltage of 70 eV), equipped with a Chrompack CP-Wax 52 CB capillary column (50 m length, 0.32 mm ID) (Chrompack, Middelburg, The Netherlands). The protocols for SPME–GC–MS analyses, and for the identification of VOCs, were previously published [15,17,18]. Identification was obtained using the NIST 11 MSMS library and the NIST MS Search Program 2.0 (NIST, Gaithersburg, MD, USA). All VOCs were relatively quantified from chromatogram peak areas and then sorted for the different chemical classes and super normalized with the mean centering method [5,6,10,17]. Quantifications of VOCS were obtained from the samples in analytical duplicates.

### 2.7. Statistical Analyses

All statistical analyses were performed using TIBCO Statistica v.8.0 (Tibco Inc., Palo Alto, CA, USA). The volatilome was checked for normality using the Shapiro–Wilk’s test and homoscedasticity with the Levene’s test [19]. Differences between all samples were evaluated with analysis of variance (ANOVA), followed by a comparison among samples and time points made with Tukey’s HSD (honestly significant difference) post hoc test (*p* < 0.05). For multivariate analyses, principal component analysis (PCA), K-means clustering, and MANOVA (multivariate ANOVA) were used to study the relationship between the variables. For multivariate analyses, the datasets were normalized using the mean-centering method [5,6,10,17]. The different datasets of results were obtained from biological duplicates of the experiment performed at different times and technical duplicates performed at the same time (*n* = 4). All results were expressed as mean values obtained at least from technical duplicates.

## 3. Results

### 3.1. Fermentation Trends

**Figure 1:**
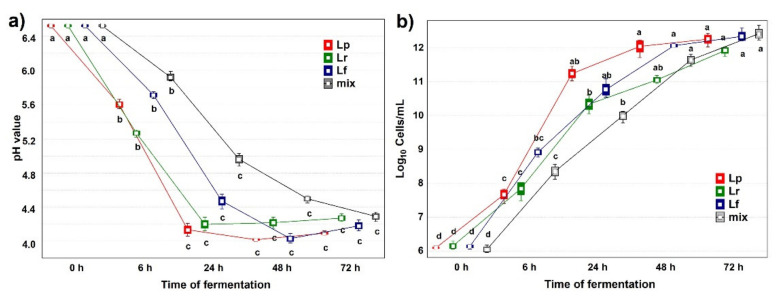
(**a**) pH values during fermentation; (**b**) bacterial cell enumeration during fermentation. ^a, b, c, d^: values with different letters within a row are significantly different by Tukey’s HSD (honestly significant difference) post hoc test *p* < 0.05. Lr = HSB fermented by *Lacticaseibacillus rhamnosus* C1112; Lp = HSB fermented by *Lactiplantibacillus plantarum* 98b; Lf = HSB fermented by *Limosilactobacillus fermentum* PRLF; m = HSB fermented by the bacterial mix of the three strains. Results shown are for 6, 24, 48, and 72 = h of fermentation.

Starting from a mean pH value of 6.55 ± 0.06, acidification was actively lasting up to 24 or 48 h and a plateau was maintained afterwards (Figure 1a). At the early time point (6 h), Lf was the inoculum that reduced the pH the most, reaching a mean value of 5.27 ± 0.04. At the intermediate time point (24 h), pH values were brought down to values ranging from 4.96 ± 0.07 (mix) to 4.13 ± 0.07 (Lp). At the late time point (48 h) pH values were brought down to values ranging from 4.49 ± 0.04 (mix) to 4.01 ± 0.02 (Lp). Similar values were then maintained up to the end point. Bacterial quantifications were expressed as mean values of plate counts and they were expressed as Log_10_ cells/mL (Figure 1b). Bacterial growth was exponential up to 24 h, and the samples were all in a range of growth spanning from means of Log_10_ cells/mL 9.99 ± 1.05 (mix) to 11.23 ± 1.34 (Lp). At the end point the samples reached a high growth ranging from means of Log_10_ cells/mL 11.92 ± 1.19 (Lr) to 12.42 ± 1.42 (mix).

### 3.2. The Volatilome over the Process 

Through SPME–GC–MS among 24 duplicated cases (*n* = 48), 213 VOCs were identified with more than 80% similarity with the NIST 11 MSMS library and the NIST MS Search Program 2.0 (NIST, Gaithersburg, MD, USA). On average, 139 were relatively quantified in the non-fermented samples and 205 were quantified during fermentation, of which 186 were found in samples fermented by Lr, 183 in samples fermented by Lp, 176 in in samples fermented by Lf, and 166 in samples fermented by the mix. A total of 173 different VOCs were detected after 6 h of fermentation, 174 after 24 h, 186 after 48 h, and 179 after 72 h. This scenario described the whole volatilome of the experiments that was expressed by normalized quantifications of peak areas from any case with an expression heatmap (Figure S2). To confer robustness to this procedure we performed statistical analyses. Among this dataset of 213 VOCs, 113 VOCs were deemed significant by ANOVA (*p* < 0.05). This new dataset was sorted by chemical class and the resulting matrices were independently normalized and expressed with PCA. Additionally, to clusterize the cases on PCA, K-means clustering analyses were performed, which identified 93 different VOCs (*p* < 0.05). This tool permitted us to describe the profile of VOCs that changed during fermentation. Moreover, with multivariate ANOVA (MANOVA) we assigned significative discrimination on 62 VOCs for the type of inoculum (Table S1) and 65 VOCs for the time of fermentation (Table S2).

#### 3.2.1. Aldehydes

**Figure 2:**
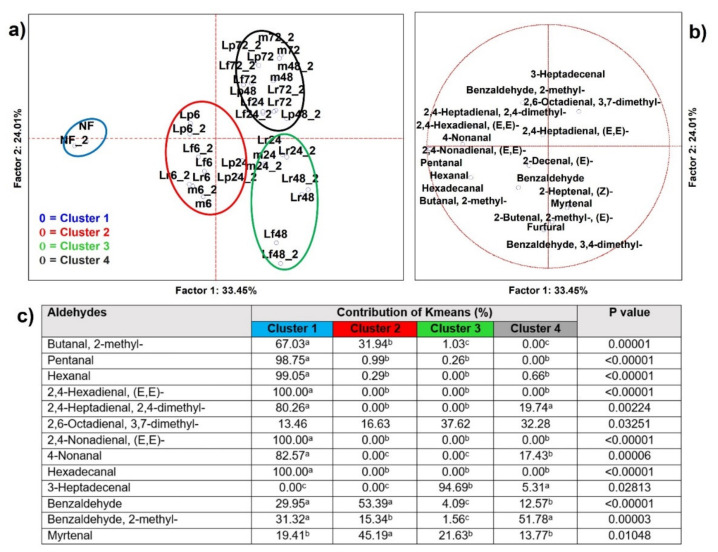
(**a**) Principal component analysis (PCA) of cases and (**b**) of variables on aldehydes (*p* < 0.05); (**c**) K-means clustering analysis (at least *p* < 0.05). NF = not fermented HSB; Lr = HSB fermented by *Lacticaseibacillus rhamnosus* C1112; Lp = HSB fermented by *Lactiplantibacillus plantarum* 98b; Lf = HSB fermented by *Limosilactobacillus fermentum* PRLF; m = HSB fermented by the bacterial mix of the three strains. Results are shown for 6, 24, 48, and 72 = h of fermentation. Blue plot = Cluster 1; red plot = Cluster 2; green plot = Cluster 3; grey plot = Cluster 4; ^a, b, c^ = values with different letters within a row are significantly different by Tukey’s HSD (honestly significant difference) post hoc test (*p* < 0.05).

From the analysis of variance that included all samples (*n* = 58), 19 aldehydes resulted as significantly different (*p* < 0.05) and 13 were able to clusterize, by K-means, the PCA cases into four sets (Figure 2), as well as address significance contributions using independent variables (MANOVA *p* < 0.01) categorized for the inoculum (Table S1) and for the time of fermentation (Table S2). Cluster 1, the smallest one, contained just unfermented HSB. It was described as the larger speciation in aldehydes (top producers of 8 out of 13 variables). In particular, pentanal, hexanal, 2,4-hexadienal (E,E), 2,4-nonadienal (E,E), and hexadecanal were exclusively features of not fermented HSB. Thus, it was evident that the aldehydes’ contribution on the final bouquet diminished over time and was not related to any fermentation. Notwithstanding, some typical features could be found in Cluster 2 where myrtenal was principally produced in the early time points by any incolum (Tables S1 and S2), or in Cluster 3 where 3-heptadecenal was attributed to *Lc. rhamnosus* (Lr) and *Lm. fermentum* (Lm) starters at the intermediate time points (Tables S1 and S2).

Aldehyde production in fermented food is a result of microbial fermentation and lipid oxidation. For instance, aliphatic linear C10–C18 aldehydes are potent odoriferous components and are applied as flavorings. Many aldehydes are required because they contribute constructively to odor and taste similar to fruity, floral, and fresh fragrances, such as myrtenal, while others are unfavorable and express a pungent aroma and are toxic at a low threshold, such as Benzaldehyde. The increased abundance of Myrtenal by the effect of fermentation must be considered promising since this compound is involved in different health related capabilities, from antimicrobial to protective effects on neurodegenerative processes [20]. Recently, it was reported that the principal species in the spontaneous fermentation of toddy palm nectar were different lactobacilli and that one of the main descriptors of the volatilome of fermentation was the bioactive VOC E-15-heptadecenal, which is probably derived from the catabolism of long-chain organic acid, such as palmitic acid [21]. Similarly, as described in Section 3.2.4., we demonstrated the presence of long chain organic acids in non-fermented HSB.

#### 3.2.2. Ketones

**Figure 3:**
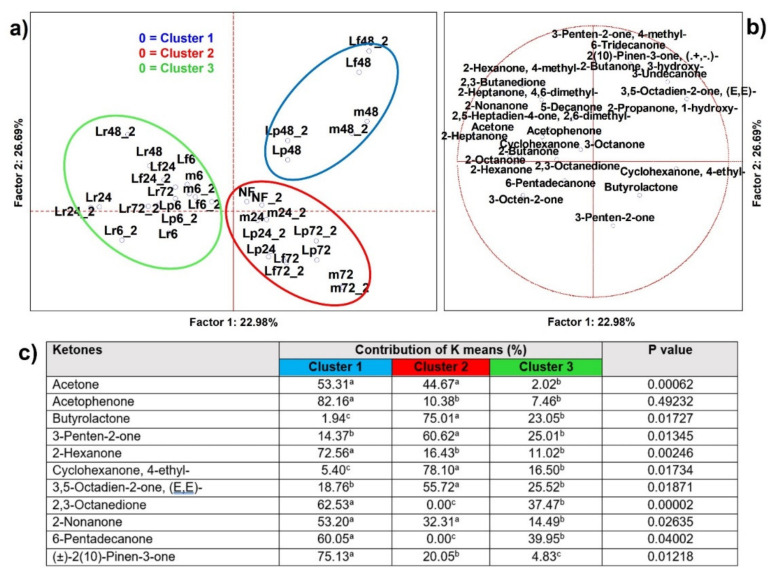
(**a**) Principal component analysis (PCA) of cases and (**b**) of variables on ketones (*p* < 0.05); (**c**) K-means clustering analysis (at least *p* < 0.05). NF = not fermented HSB; Lr = HSB fermented by *Lacticaseibacillus rhamnosus* C1112; Lp = HSB fermented by *Lactiplantibacillus plantarum* 98b; Lf = HSB fermented by *Limosilactobacillus fermentum* PRLF; m = HSB fermented by the bacterial mix of the three strains. Results shown are for 6, 24, 48, and 72 = h of fermentation. Blue plot = Cluster 1; red plot = Cluster 2; green plot = Cluster 3; ^a, b, c^ = values with different letters within a row are significantly different by Tukey’s HSD (honestly significant difference) post hoc test (*p* < 0.05).

From the analysis of variance including all samples (*n* = 58), 27 ketones resulted as significantly different (*p* < 0.05) and 11 were able to clusterize, by K-means, the PCA cases into three sets (Figure 3), as well as address the significance contributions by independent variables (MANOVA *p* < 0.01) categorized for the inoculum (Table S1) and for the time of fermentation (Table S2). Cluster 1 was made by the 48 h cases from any fermentation starter, except fot *Lc. rhamnosus*. The few significant VOCs that described this set were acetophenone, (±)-2(10)-pinen-3-one, known as pinocarvone, and 2-nonanone. Cluster 2 was composed of the late time point cases, especially those related to *Lp. plantarum* (Lp), *Lm. fermentum*, and the bacterial mix (m), whose descriptors were 3-penten-2-one and butyrolactone. Lastly, Cluster 3 included fermented samples with *Lc. rhamnosus*, but no variables were heavier than other clusters.

There was a low number of ketones that were able to discriminate whether the cases indicated were associated to the fermentation process, regardless of the starter used; in fact just two out of eleven species were able to discriminate the samples before fermentation (Table S2). Ketone production was a result of bacterial fermentation and lipid oxidation. Some of them were desirable, such as acetophenone, pinocarvone, or butyrolactone that, as bioactive compounds, had antioxidant or antimicrobial potential and were produced by lactic acid bacteria during degradation of lignins or deconjugation of polyphenols [22,23,24,25], and 2-nonanone, that has been described to confer several positive sensory/aroma attributes. Recently, another work has described positive correlations among the increased quantity of acetophenone and the abundance of lactobacilli after fermentation of a fiber rich in phenolic compounds [26]. 2-nonanone has been found in plants, e.g., cinnamon, cloves, and coconut, in which it showed insecticidal activity in addition to contributing to the flavor [5]. Besides, it was found to be a descriptor of dairy products with a higher storage time when fermented by *Lp. plantarum* P-8 [27]. In another work, it was found to be a descriptor of oat fermented by *Lacticaseibacillus paracasei*, where it was probably derived from the unsaturated lipids present in oat [28].

#### 3.2.3. Alcohols

**Figure 4:**
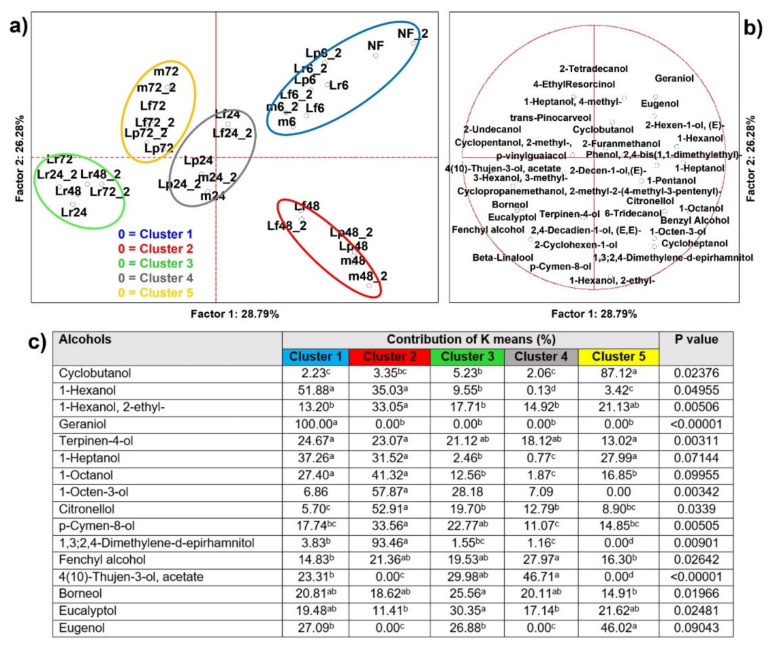
(**a**) Principal component analysis (PCA) of cases and (**b**) of variables on alcohols (*p* < 0.05); (**c**) K-means clustering analysis (at least *p* < 0.05). NF = not fermented HSB; Lr = HSB fermented by *Lacticaseibacillus rhamnosus* C1112; Lp = HSB fermented by *Lactiplantibacillus plantarum* 98b; Lf = HSB fermented by *Limosilactobacillus fermentum* PRLF; m = HSB fermented by the bacterial mix of the three strains. The results shown are for 6, 24, 48, and 72 = h of fermentation. Blue plot = Cluster 1; red plot = Cluster 2; green plot = Cluster 3; grey plot = Cluster 4; yellow plot = Cluster 5; ^a, b, c, d^ = values with different letters within a row are significantly different by Tukey’s HSD (honestly significant difference) post hoc test (*p* < 0.05).

From analysis of variance including all samples (*n* = 34), 37 alcohols resulted as significantly different (*p* < 0.05) and 22 were able to clusterize, by K-means, the PCA loadings into five sets (Figure 4), as well as address significance contributions by independent variables (MANOVA *p* < 0.01) categorized for the inoculum (Table S1) and for the time of fermentation (Table S2). Cluster 1 was made by the poorly fermented and not fermented cases and geraniol was its signature (Table S1). Cluster 2 included different fermenting strains but only 48 h cases were present, and it was the sole producer of 1,3;2,4-dimethylene-d-epirhamnitol, although it even was described by citronellol and 1-octen-3-ol. Cluster 3 included 24 h cases and it was described mainly by 4(10)-thujen-3-ol acetate (Table S2). Cluster 4 comprised no 72 h cases, except those fermented by *Lc. rhamnosus*, and was described principally by cyclobutanol, eugenol, p-vinylguaiacol, and beta-Linalool. Cluster 5, interestingly, was specifically associated to the fermentation using strain *Lc. Rhamnosus*, whatever the time point, of eleven different alcohols (MANOVA *p* < 0.01) (Table S1). Besides their general role as main products of fermentation, they contributed to improve microbial stability and food quality. Moreover eugenol, beta-linalool, and citronellol are considered bioactive molecules and were reported to modulate beneficially the gut microbiome [5,24,25,29] and to possess anti-inflammatory and antioxidant activities [30,31,32,33]. Furthermore, they owned a pleasant fruity and fresh nuance. 1-octen-3-ol was derived from linoleic acid during oxidative breakdown, and it had antimicrobial activity against spoilage and opportunistic microbes [34]. 1-octen-3-ol was found to be one of the main contributors to oat samples fermented by *Lc. paracasei* [28]. Their increased abundance during fermentation has to be considered as an increase in quality and functional food potential. 

#### 3.2.4. Organic Acids

**Figure 5:**
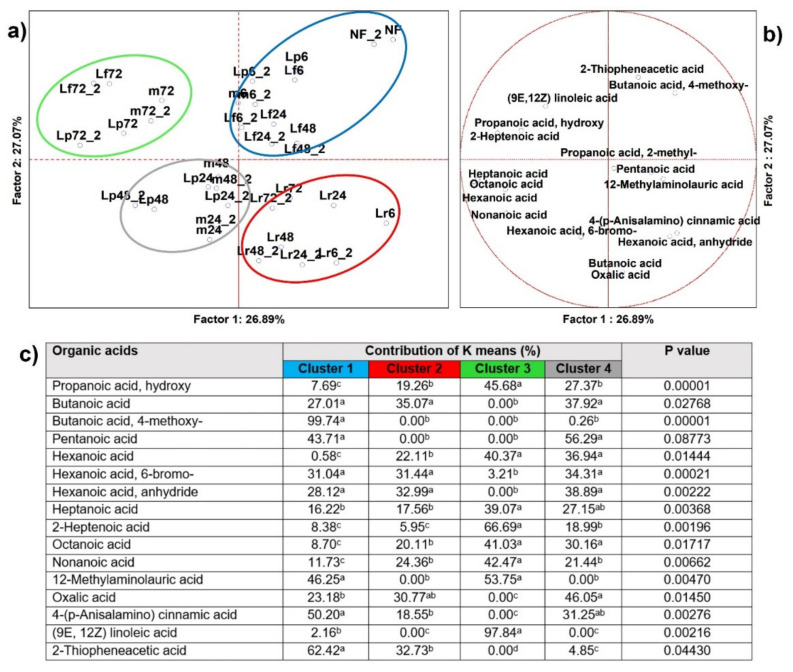
(**a**) Principal component analysis (PCA) of cases and (**b**) of variables on organic acids (*p* < 0.05); (**c**) K-means clustering analysis (at least *p* < 0.05). NF = not fermented HSB; Lr = HSB fermented by *Lacticaseibacillus rhamnosus* C1112; Lp = HSB fermented by *Lactiplantibacillus plantarum* 98b; Lf = HSB fermented by *Limosilactobacillus fermentum* PRLF; m = HSB fermented by the bacterial mix of the three strains. Results shown are from 6, 24, 48, and 72 = h of fermentation. Blue plot = Cluster 1; red plot = Cluster 2; green plot = Cluster 3; grey plot = Cluster 4; ^a, b, c, d^ = values with different letters within a row are significantly different by Tukey’s HSD (honestly significant difference) post hoc test (*p* < 0.05).

From analysis of variance including all samples (*n* = 34), 18 organic acids resulted as significantly different (*p* < 0.05) and 16 were able to clusterize, by K-means, the PCA loadings into four sets (Figure 5), as well to address significance contributions by independent variables (MANOVA *p* < 0.01) categorized for the inoculum (Table S1) and for the time of fermentation (Table S2). Members of Cluster 1 were those cases poorly fermented that were set along non fermented BH and were described principally by 2-thiopheneacetic acid, butanoic acid, and 4-methoxy. Cluster 2 comprised any case fermented by *Lc. rhamnosus* without regard to time points (Table S1) and was described by a mild contribution to the organic acid basket with no exclusive sign except noteworthy production of butanoic acid. Cluster 3 included intermediate time point cases of the pool and *Lp. plantarum* and was described by pentanoic acid to whose production contributed the most. Cluster 4 was instead related to the late-time points (Table S2) of any of the four starters, and had the larger speciation of organic acids, as well as the major contributions of 8 out of 16 dependent variables, mainly propanoic acid hydroxy, (9E,12Z), linoleic acid, and 2-heptenoic acid.

In our study, the complexity, quality, and quantity of the profile of organic acids increased over time. At the late time point the most important bioactive compounds were produced, e.g., short and medium chain fatty acids, as well as those that contributed to the aroma (Table S2). In agreement with previous studies [10,35], the increased concentration of propanoic, lactic, and medium-chain organic acids depended mainly on the time of fermentation rather than by the lactobacilli starter. Butanoic and lactic acids are used in the food industry as flavors, and as nutritional and antimicrobial compounds that contribute largely to improve the quality and safety of fermented foods [36,37]. In addition, butanoic acid fits the new definition of prebiotics [38,39]. Among medium chain fatty acids, they all bear health-related attributes [40,41], but some have unpleasant off-odors. For example, nonanoic acid may represent a marker compound of hemp seed product, owning a typical scent [10,35], hexanoic and octanoic acids possess rancid-like traits [42,43] and their concentrations should be modulated in the food product. Lactic acid bacteria are used to produce short- and medium-chain organic acids from fiber degradation [44]. These organic acids are probably derived from the alcohols and polyols produced via fermentation of fiber monomer carbohydrates. It is reported that lactobacilli are able to produce hexanoic acid from D-glucitol as the final product of lactose fermentation [45].

#### 3.2.5. Alkenes

**Figure 6:**
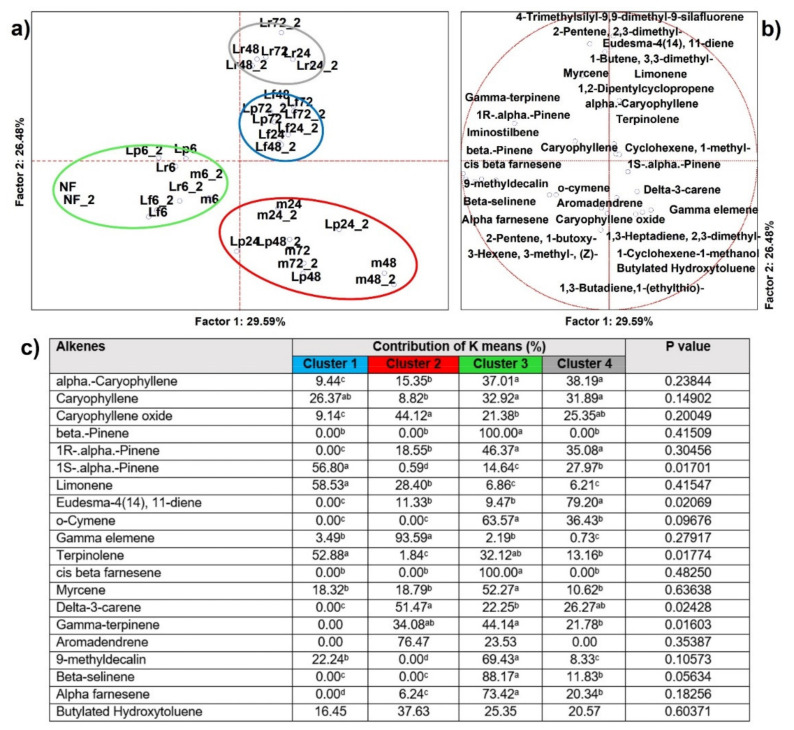
(**a**) Principal component analysis (PCA) of cases and (**b**) of variables on alkenes (*p* < 0.05); (**c**) K-means clustering analysis (at least *p* < 0.05). NF = not fermented HSB; Lr = HSB fermented by *Lacticaseibacillus rhamnosus* C1112; Lp = HSB fermented by *Lactiplantibacillus plantarum* 98b; Lf = HSB fermented by *Limosilactobacillus fermentum* PRLF; m = HSB fermented by the bacterial mix of the three strains. Results shown are for 6, 24, 48, and 72 = hours of fermentation. Blue plot = Cluster 1; red plot = Cluster 2; green plot = Cluster 3; grey plot = Cluster 4; ^a, b, c, d^ = values with different letters within a row are significantly different by Tukey’s HSD (honestly significant difference) post hoc test (*p* < 0.05).

From analysis of variance including all samples (*n* = 34), 32 alkenes resulted as significantly different (*p* < 0.05) and 22 were able to clusterize, by K-means, the PCA cases into four sets (Figure 6), as well as address significance contributions by independent variables (MANOVA *p* < 0.01) categorized for the inoculum (Table S1) and for the time of fermentation (Table S2). Cluster 1 included members of *Lm. fermentum* and *Lc. rhamnosus* fermentations at the intermediate time points and were described by three specific features, i.e., terpinolene, limonene, and 1(S)-alpha-pinene. Cluster 2 was composed by any case of the pool after 6 h of fermentation and was described to be rich in gamma-elemene and aromadendrene. Cluster 3 contained the unfermented HSB and the early time points of the fermentations and was described by the larger speciation in alkenes and two exclusive signatures, such as beta-pinene and cis-beta-farnesene. Cluster 4 was again harboring just the *Lc. rhamnosus* fermented cases at the late time points, but was described significantly by few variables, in particular eudesma 4-(14) and 11-diene, which accounted for almost 80% of the production (MANOVA *p* < 0.01) (Table S1). Alkenes are a class of chemical compounds with a broad nature and capacity, from this dataset many of them are the characteristic terpenes found in hemp seed oil with fantastic health-related activities [46,47]. Notwithstanding, they contribute chiefly to the generation of a desirable aromatic bouquet, possessing traits of fresh, green, floral, and fruity nuances, such as beta-pinene, cis-beta-farnesene, aromadendrene, and myrcene [48]. In our work, the alkenes that were descriptors of fermentations were probably due to bioconversion of phenolic compounds, as it is known that under the action of *Lactobacillaceae* phenols are transformed, liberating alkenes and producing alcohols [49].

#### 3.2.6. Amines

**Figure 7:**
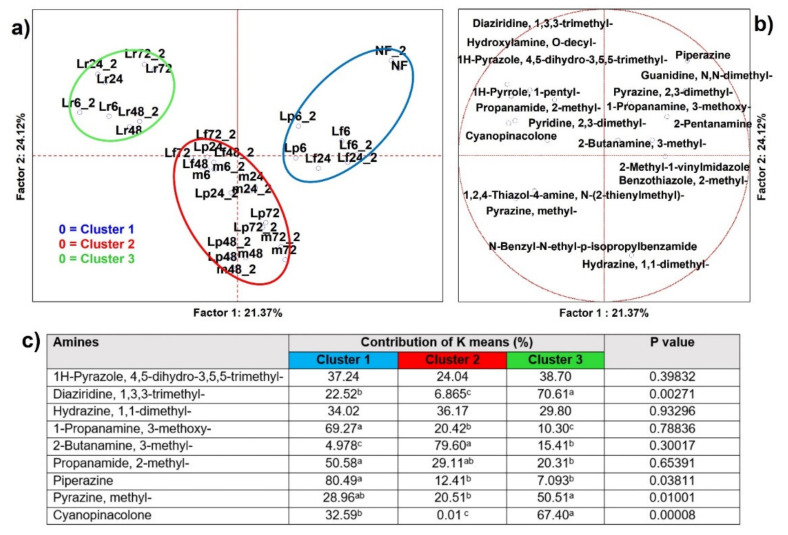
(**a**) Principal component analysis (PCA) of cases and (**b**) of variables on amines (*p* < 0.05); (**c**) K-means clustering analysis (at least *p* < 0.05). NF = not fermented HSB; Lr = HSB fermented by *Lacticaseibacillus rhamnosus* C1112; Lp = HSB fermented by *Lactiplantibacillus plantarum* 98b; Lf = HSB fermented by *Limosilactobacillus fermentum* PRLF; m = HSB fermented by the bacterial mix of the three strains. Results shown are for 6, 24, 48, and 72 = h of fermentation. Blue plot = Cluster 1; red plot = Cluster 2; green plot = Cluster 3; ^a, b, c^ = values with different letters within a row are significantly different by Tukey’s HSD (honestly significant difference) post hoc test (*p* < 0.05).

From analysis of variance including all samples (*n* = 34), 20 amines resulted as significantly different (*p* < 0.05) and 9 were able to clusterize, by K-means, the PCA cases in three sets (Figure 7), as well as address significance contributions by independent variables (MANOVA *p* < 0.01) categorized for the inoculum (Table S1) and for the time of fermentation (Table S2). Cluster 1 included members. Cluster 1 included unfermented or early-time fermented cases, with the former set almost apart and described by undesirable amines for food applications, such as piperazine, 2-pentanamine, propanamine, and 3-methoxy, that in certain concentration could result as toxic [50,51], and anyhow are known to generate strong and unpleasant odors. For example, most of the amines have an odor generally described as fishy, rotten egg-like, skunk-like, and penetrating, but piperazine has a scent described as musty and dirty smelling that is so strong it overrides the previous scents [52]. Cluster 2 is that including almost all cases that fermented longer than 6 h, other than the *Lc. rhamnosus* ones, that were grouped in Cluster 3. Interestingly, these two groups were described by a minor speciation of amines, as well as a minor contribution on the production of amines (MANOVA *p* < 0.01) (Tables S1 and S2). 

## 4. Conclusions

HSB is an untapped byproduct of the hemp industrial food sector that is nutritionally superior but with an unfriendly and pungent taste that makes its application in food products difficult.

The main culprits of the typical unpleasant odor of hemp seed derivates are mainly the amines, such as putrescine and piperazine. Even if the fresh, fruity, and bloomy nuances of the terpenes bouquet, as well as the citrus and almond breezes generated by alcohols and aldehydes, are used to cover the amines’ stinky odor, their content is reduced with processing, resulting in the masking effect of the pleasant-smelling profile, and the nasty taste of the amines returning to the foods. From our results, it seems that fermentation with beneficial lactobacilli could reduce and control the problem since they are able to reduce the content of the unpleasant VOCs that have historically limited the palatability of hemp seed foods. Additionally, we have demonstrated that with lactobacilli fermentation the abundance of other VOCs with bioactivity and health-related (direct or secondary) actions are augmented (e.g., hexanoic acid) or are implemented by the transformation of poorly bio-disponible complex compounds (e.g., 1-octen-3-ol). From the recipient investigation we have found that the time and the type of starter employed in fermentations could be adjusted relatively to tune the right balance of bioactive and nutritional compounds in the final product, as well as to modulate the aromatic profile, considering either scents or tastes of the final product.

## Data Availability

Not applicable.

## Supplementary Materials

The following are available online at https://www.mdpi.com/article/10.3390/microorganisms9122418/s1, Figure S1: Calibration curves for the spectrophotometric evaluation (Optical Density at 600 nm) of bacterial cell loads (CFU/mL); Figure S2: Heatmap of the whole volatilome of the experiment; Table S1: MANOVA (*p* < 0.01) categorized for the bacterial inocula and % of contribution to discrimination and Table S2: MANOVA (*p* < 0.01) categorized for the time points of fermentation and % of contribution to discrimination.
