# Supplementary material for: The Exploitation of a Hempseed Byproduct to Produce Flavorings and Healthy Food Ingredients by a Fermentation Process
Source: Microorganisms. 2021 Nov 23;9(12):2418. doi: 10.3390/microorganisms9122418 (PMC8707447; doi:10.3390/microorganisms9122418)
Supplement: Supplementary file 1 [file microorganisms-09-02418-s001.zip › microorganisms-1458426-supplementary.pdf]

microorganisms

Article

The Exploitation of a Hempseed Byproduct to Produce Flavorings and Healthy Food Ingredients by a Fermentation Process

Lorenzo Nissen [1,2], Flavia Casciano [2], Elena Babini [1,3] and Andrea Gianotti [1,2,3,*]

[1] CIRI (Interdepartmental Centre of Agri-Food Industrial Research), *Alma Mater Studiorum* - University of Bologna, P.za Goidanich 60, 47521 Cesena, Italy; lorenzo.nissen@unibo.it (L.N.); elena.babini2@unibo.it (E.B.)
[2] DISTAL (Department of Agricultural and Food Sciences), *Alma Mater Studiorum* - University of Bologna, V. le Fanin 44, 40127, Bologna, Italy; flavia.casciano2@unibo.it
[3] DISTAL (Department of Agricultural and Food Sciences), Campus of Food Sciences, *Alma Mater Studiorum* - University of Bologna, P.za Goidanich 60, 47521, Cesena, Italy
* Correspondence: andrea.gianotti@unibo.it

Supplementary Material

Table S1. MANOVA (*P* < 0.01) categorized for the bacterial inocula and % of contribution to discrimination.

VOCs	Type of Inoculum				
	NF*	C1112 (Lc)	98b (Lp)	PRLF (Lr)	mix (m)
**Aldehydes**					
Hexanal	99.04[a]	0.04[b]	0.75[b]	0.00[b]	0.16[b]
2,4-Hexadienal, (E,E)-	100.00[a]	0.00[b]	0.00[b]	0.00[b]	0.00[b]
2,4-Heptadienal, 2,4-dimethyl-	77.49[a]	0.00[c]	0.00[c]	22.51[b]	0.00[c]
2,6-Octadienal, 3,7-dimethyl-	9.47[c]	40.16[a]	23.85[b]	9.00[c]	17.52[b]
2,4-Nonadienal, (E,E)-	100.00[a]	0.00[b]	0.00[b]	0.00[b]	0.00[b]
4-Nonanal	80.45[a]	0.00[c]	19.55[b]	0.00[c]	0.00[c]
2-Decenal, (E)-	33.69[b]	0.00[c]	0.00[c]	58.86[a]	7.45[c]
3-Heptadecenal	0.00[b]	0.00[b]	2.60[b]	3.93[b]	93.47[a]
Myrtenal	20.90[b]	11.38[c]	10.84[c]	49.09[a]	7.79[c]
**Alcohols**					
Geraniol	91.19[a]	8.81[b]	0.00[c]	0.00[c]	0.00[c]
Terpinen-4-ol	8.64[b]	31.66[a]	23.83[a]	11.75[b]	24.13[a]
Citronellol	9.26[b]	13.77[b]	21.00[a]	21.00[a]	34.97[a]
Beta-Linalool	7.70[b]	34.22[a]	23.43[a]	7.48[b]	27.17[a]
p-Cymen-8-ol	8.95[b]	27.59[a]	26.69[a]	9.45[b]	27.31[a]
Fenchyl alcohol	0.00[b]	27.34[a]	23.51[a]	23.40[a]	25.75[a]
4(10)-Thujen-3-ol, acetate	10.05[b]	57.11[a]	19.89[b]	12.95[b]	0.00[c]
Borneol	1.61[c]	49.17[a]	19.57[b]	8.67[c]	20.98[b]
Eucalyptol	7.69[b]	30.69[a]	25.92[a]	12.46[b]	23.24[a]
trans-Pinocarveol	0.00[c]	25.31[a]	9.69[b]	33.16[a]	31.84[a]
Phenol, 2,4-bis(1,1-dimethylethyl)-	39.16[a]	7.57[c]	16.83[b]	9.63[bc]	26.82[a]
4-EthylResorcinol	68.26[a]	14.26[b]	11.30[b]	4.02[c]	2.17[c]
**Ketones**					
Acetone	0.00[c]	61.44[a]	16.35[b]	11.18[b]	11.02[b]
Acetophenone	0.00[c]	31.03[a]	25.31[a]	15.63[b]	28.03[a]
2-Propanone, 1-hydroxy-	0.00[c]	9.59[b]	8.92[b]	34.27[a]	47.22[a]
2,3-Butanedione	0.54[d]	50.09[a]	11.33[c]	27.81[b]	10.22[c]
Butyrolactone	0.00[c]	12.13[b]	32.48[a]	12.68[b]	42.71[a]
2-Hexanone	25.26[b]	45.73[a]	1.41[c]	27.25[b]	0.35[c]
cyclohexanone, 4-ethyl-	0.00[d]	45.00[a]	29.35[b]	6.83[c]	18.81[b]
2,5-Heptadien-4-one, 2,6-dimethyl-	2.47[b]	24.56[a]	29.39[a]	22.73[a]	20.85[a]
3,5-Octadien-2-one, (E,E)-	33.42[a]	9.33[b]	17.31[b]	28.27[a]	11.66[b]

6-Pentadecanone	0.00^{c}	32.71^{b}	67.29^{a}	0.00^{c}	0.00^{c}
Pinocarvone	0.00^{d}	8.32^{c}	4.47^{cd}	50.78^{a}	36.44^{b}
**Organic Acids**					
Propanoic acid, hydroxy	4.47	9.33	39.77	31.43	15.00
Butanoic acid, 4-methoxy-	70.79	6.51	4.46	8.21	10.04
Hexanoic acid, 6-bromo-	0.00	22.21	25.08	23.43	29.29
Hexanoic acid, anhydride	43.72	56.28	0.00	0.00	0.00
Heptanoic acid	0.00	15.81	29.42	25.13	29.64
2-Heptenoic acid	0.00	10.53	55.50	21.75	12.22
12-Methylaminolauric acid	70.59	29.41	0.00	0.00	0.00
Oxalic acid	0.00	51.26	25.02	3.77	19.95
4-(p-Anisalamino) cinnamic acid	0.00	43.11	17.07	20.29	19.52
(9E,12Z) Linoleic acid	0.00	0.00	0.00	33.95	66.05
**Alkenes**					
alpha-Caryophyllene	21.91	52.29	12.70	13.10	0.00
Caryophyllene	16.28	43.47	14.42	10.77	15.06
Iminostilbene	100.00	0.00	0.00	0.00	0.00
o-cymene	55.05	0.00	18.68	26.27	0.00
Gamma elemene	10.19	2.20	1.29	0.00	86.32
cis beta farnesene	98.02	1.98	0.00	0.00	0.00
Delta-3-carene	5.33	22.99	22.30	3.94	45.44
Gamma-terpinene	57.95	20.14	12.77	9.15	0.00
Aromadendrene	67.24	0.00	2.55	4.39	25.82
9-methyldecalin	76.33	6.85	9.06	0.00	7.76
Beta-selinene	83.39	4.15	6.65	0.00	5.81
Alpha farnesene	94.19	2.50	3.30	0.00	0.00
Butylated Hydroxytoluene	0.00	30.31	26.21	18.50	24.97
**Amines**					
Piperazine	86.40^{a}	13.02^{c}	0.58^{b}	0.00^{d}	0.00^{d}
1H-Pyrazole, 4,5-dihydro-3,5,5-trimethyl-	0.00	46.27	19.84	25.13	8.76
Diaziridine, 1,3,3-trimethyl-	0.00	53.34	16.06	22.76	7.84
Hydrazine, 1,1-dimethyl-	0.00	4.55	53.91	22.13	19.41
1-Propanamine, 3-methoxy-	72.62	6.42	8.19	9.61	3.16
2-Butanamine, 3-methyl-	35.04	16.27	2.61	3.83	42.26
N-Benzyl-N-ethyl-p-isopropylbenzamide	2.07	17.58	34.91	21.71	23.73
Pyrazine, methyl-	1.07	28.75	24.16	19.04	26.98
Cyanopinacolone	0.00	47.64	20.37	19.74	12.25

$^{a, b, c, d}$ Values with different letters within a row are significantly different by Tukey's HSD (honestly significant difference) post hoc test $P < 0.05$. *NF = not fermented samples; C1112 = *Lacticaseibacillus rhamnosus* C1112; 98b = *Lactiplantibacillus plantarum* 98b; PRLF = *Limosilactobacillus fermentum* PRLF; mix = the bacterial mix of the three strains.

Table S2. MANOVA ($P < 0.01$) categorized for the time points of fermentation and % of contribution to discrimination.

VOCs	Time of Fermentation (h)				
	**0 h**	**6 h**	**24 h**	**48 h**	**72 h**
**Aldehydes**					
Butanal, 2-methyl-	68.75^{a}	26.37^{b}	0.00^{d}	0.00^{d}	4.89^{cd}
Hexanal	99.02^{a}	0.98^{b}	0.00^{b}	0.00^{b}	0.00^{b}
2,4-Hexadienal, (E,E)-	100.00^{a}	0.00^{b}	0.00^{b}	0.00^{b}	0.00^{b}
2,4-Heptadienal, 2,4-dimethyl-	73.35^{a}	0.00^{c}	0.00^{c}	0.00^{c}	26.65^{b}
2,4-Nonadienal, (E,E)-	100.00^{a}	0.00^{b}	0.00^{b}	0.00^{b}	0.00^{b}
4-Nonanal	80.45^{a}	19.55^{b}	0.00^{c}	0.00^{c}	0.00^{c}
Myrtenal	9.89^{b}	31.90^{a}	45.42^{a}	8.16^{b}	4.63^{b}
Benzaldehyde	32.27^{a}	54.01^{a}	9.86^{b}	1.29^{b}	2.57^{b}
Benzaldehyde, 3,4-dimethyl-	8.98^{b}	39.08^{a}	14.37^{ab}	28.97^{a}	8.59^{b}
**Alcohols**					
Geraniol	91.22^{a}	8.78^{b}	0.00^{c}	0.00^{c}	0.00^{c}
Terpinen-4-ol	10.18^{b}	20.72^{a}	23.36^{a}	24.94^{a}	20.80^{a}
Citronellol	9.26^{b}	6.52^{b}	7.77^{b}	37.66^{a}	38.78^{a}

Beta-Linalool	7.70^{c}	22.42^{ab}	32.85^{a}	19.75^{b}	17.28^{b}
2-Decen-1-ol,(E)-	6.26^{b}	8.18^{b}	32.84^{a}	42.36^{a}	10.35^{b}
2,4-Decadien-1-ol, (E,E)-	0.00^{c}	2.36^{c}	16.61^{b}	75.05^{a}	5.98^{bc}
p-Cymen-8-ol	8.95^{b}	22.06^{a}	25.23^{a}	24.97^{a}	18.78^{a}
1,3;2,4-Dimethylene-d-epirhamnitol	0.00^{c}	9.72^{b}	6.60^{b}	83.68^{a}	0.00^{c}
Fenchyl alcohol	0.00^{b}	22.49^{a}	27.46^{a}	26.74^{a}	23.30^{a}
4(10)-Thujen-3-ol, acetate	10.07^{c}	22.70^{b}	38.04^{a}	11.10^{c}	18.09^{bc}
Borneol	1.61^{b}	27.39^{a}	29.48^{a}	22.82^{a}	18.70^{a}
Eucalyptol	7.69^{c}	22.53^{ab}	33.95^{a}	13.58^{b}	22.24^{ab}
Eugenol	76.55^{a}	0.00^{c}	0.00^{c}	0.00^{c}	23.45^{b}
trans-Pinocarveol	2.66^{c}	26.55^{ab}	15.28^{b}	34.56^{a}	20.95^{ab}
p-vinylguaiacol	0.00^{d}	4.24^{c}	74.72^{a}	20.89^{b}	0.15^{d}
4-EthylResorcinol	68.26^{a}	13.03^{b}	9.70^{bc}	5.71^{cd}	3.30^{d}
**Ketones**					
Acetone	0.00^{c}	59.21^{a}	11.28^{b}	19.37^{b}	10.14^{b}
Acetophenone	0.00^{b}	38.86^{a}	0.00^{b}	36.06^{a}	25.08^{a}
2-Butanone, 3-hydroxy-	0.00^{d}	0.46^{d}	31.48^{b}	57.17^{a}	10.89^{c}
Butyrolactone	0.00^{b}	0.00^{b}	0.00^{b}	41.12^{a}	58.88^{a}
2-Hexanone, 4-methyl-	0.00^{b}	25.27^{a}	28.86^{a}	27.61^{a}	18.26^{a}
2-Heptanone, 4,6-dimethyl-	0.00^{c}	31.29^{a}	22.19^{ab}	27.94^{a}	18.59^{b}
2,5-Heptadien-4-one, 2,6-dimethyl-	2.47^{c}	31.16^{a}	22.09^{ab}	26.20^{ab}	18.08^{b}
2,3-Octanedione	0.00^{c}	12.60^{b}	87.40^{a}	0.00^{c}	0.00^{c}
6-Pentadecanone	0.00^{c}	28.41^{b}	71.59^{a}	0.00^{c}	0.00^{c}
Pinocarvone	0.00^{d}	0.00^{d}	23.23^{b}	74.52^{a}	2.24^{c}
**Organic acids**					
Propanoic acid, hydroxy	11.96^{c}	0.85^{d}	11.23^{c}	20.00^{b}	55.95^{a}
Butanoic acid, 4-methoxy-	93.75^{a}	0.00^{c}	0.00^{c}	6.25^{b}	0.00^{c}
Hexanoic acid, 6-bromo-	0.00^{c}	0.00^{c}	0.00^{c}	12.34^{b}	87.66^{a}
Hexanoic acid, anhydride	11.01^{c}	41.13^{a}	26.57^{b}	12.57^{c}	8.72^{c}
Heptanoic acid	36.72^{a}	7.53^{c}	12.12^{bc}	20.00^{b}	23.63^{b}
2-Heptenoic acid	18.69^{b}	7.23^{c}	0.00^{d}	18.55^{b}	55.53^{a}
12-Methylaminolauric acid	28.17^{a}	28.17^{a}	10.18^{b}	10.95^{b}	22.55^{a}
Oxalic acid	23.48^{a}	25.25^{a}	22.46^{a}	20.00^{a}	8.81^{b}
4-(p-Anisalamino) cinnamic acid	4.08^{c}	3.83^{c}	19.92^{b}	63.77^{a}	8.40^{b}
(9E,12Z) Linoleic acid	0.00^{a}	13.16^{b}	13.16^{b}	13.16^{b}	60.52^{a}
**Alkenes**					
beta-Pinene	78.16^{a}	21.84^{b}	0.00^{c}	0.00^{c}	0.00^{c}
Iminostilbene	85.99^{a}	14.01^{b}	0.00^{c}	0.00^{c}	0.00^{c}
Terpinolene	7.40^{b}	40.40^{a}	52.21^{a}	0.00^{c}	0.00^{c}
cis beta farnesene	90.22^{a}	7.96^{b}	1.82^{c}	0.00^{d}	0.00^{d}
Delta-3-carene	5.33^{b}	35.52^{a}	28.61^{a}	25.04^{a}	5.49^{b}
Gamma-terpinene	57.95^{a}	21.76^{b}	4.25^{c}	16.04^{b}	0.00^{d}
Aromadendrene	65.68^{a}	33.16^{b}	0.58^{c}	0.58^{c}	0.00^{d}
9-methyldecalin	76.33^{a}	14.67^{b}	6.88^{c}	2.12^{d}	0.00^{e}
Beta-selinene	83.39^{a}	7.61^{b}	4.86^{b}	4.15^{b}	0.00^{c}
Alpha-farnesene	94.19^{a}	2.50^{b}	1.96^{c}	0.65^{d}	0.70^{d}
2-Pentene, 2,3-dimethyl-	0.67^{b}	5.83^{b}	2.23^{b}	91.28^{a}	0.00^{c}
1-Cyclohexene-1-methanol	5.58^{c}	10.85^{b}	83.43^{a}	0.14^{d}	0.00^{d}
4-Trimethylsilyl-9,9-dimethyl-9-silafluorene	0.00^{d}	0.00^{d}	4.20^{c}	57.47^{a}	38.33^{b}
Butylated Hydroxytoluene	0.00^{b}	0.00^{b}	35.94^{a}	30.67^{a}	33.39^{a}
**Amines**					
Piperazine	90.44^{a}	3.00^{c}	6.57^{b}	0.00^{d}	0.00^{d}
1H-Pyrazole, 4,5-dihydro-3,5,5-trimethyl-	0.00^{d}	25.99^{b}	44.21^{a}	11.25^{c}	18.55^{bc}
Hydrazine, 1,1-dimethyl-	0.00^{d}	8.54^{c}	18.64^{b}	55.98^{a}	16.84^{b}
1-Propanamine, 3-methoxy-	72.62^{a}	1.64^{c}	7.36^{b}	7.76^{b}	10.62^{b}
Propanamide, 2-methyl-	8.64^{c}	28.63^{b}	62.73^{a}	0.00^{d}	0.00^{d}
Pyrazine, 2,3-dimethyl-	75.72^{a}	4.89^{c}	0.47^{d}	2.14^{cd}	16.78^{b}
Pyrazine, methyl-	1.07^{c}	31.08^{a}	22.68^{ab}	27.22^{ab}	17.95^{b}

$^{a, b, c, d}$ Values with different letters within a row are significantly different by Tukey's HSD (honestly significant difference) post hoc test ($P < 0.05$).

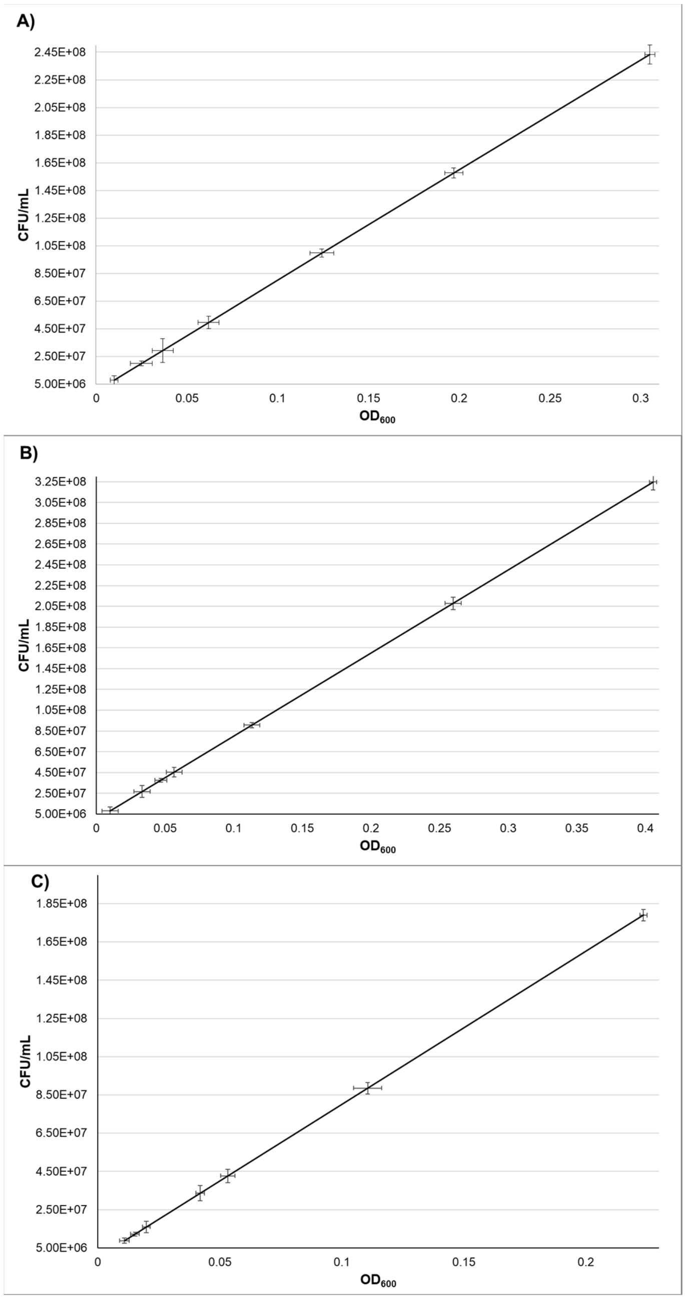

Figure S1. Calibration curves for the spectrophotometric evaluation (Optical Density at 600 nm) of bacterial cell loads (CFU/mL). (**A**) Lr = HSB fermented by *Lacticaseibacillus rhamnosus* C1112; (**B**) Lp = HSB fermented by *Lactiplantibacillus plantarum* 98b; (**C**) Lf = HSB fermented by *Limosilactobacillus fermentum* PRLF.

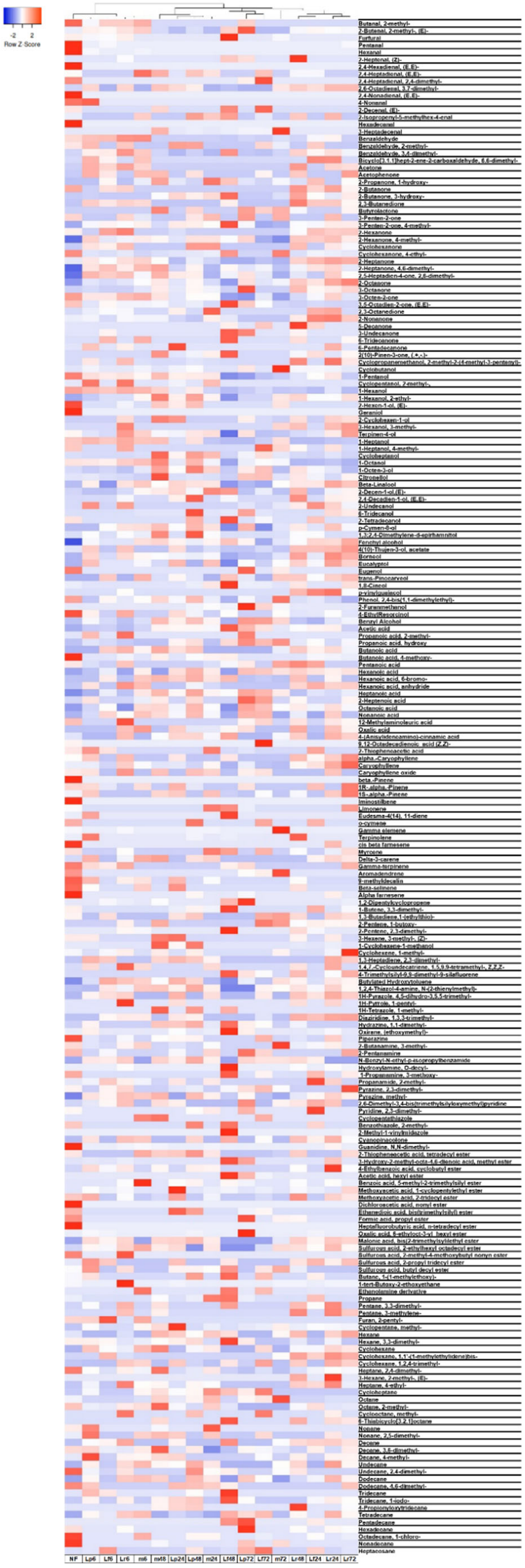

Figure S2. Heatmap of the whole volatilome of the experiment.
